# Biological investigations in Indian psychiatry

**DOI:** 10.4103/0019-5545.69225

**Published:** 2010-01

**Authors:** Rishikesh V. Behere, Naren P. Rao, Ganesan Venkatasubramanian

**Affiliations:** Department of Psychiatry, National Institute of Mental Health and Neurosciences, Bangalore - 560 029, India

**Keywords:** Blood investigations, EEG, Neuro-imaging

## Abstract

The biological basis of psychiatric disorders has been the focus of research in various studies across the world including India. In this selective overview we summarize findings of various EEG, neuro-imaging and blood related studies that have been reported in the Indian Journal of Psychiatry.

## INTRODUCTION

The biological basis for psychiatric disorders has been increasingly becoming a major research focus over the past many decades in various countries. Similar trends have been observed in Indian psychiatry too. In this selective overview, we have attempted to summarize various biological investigations that have been reported in the Indian J Psychiatry over the past many decades

## SCHIZOPHRENIA

Studies involving schizophrenia subjects have primarily looked at blood levels of various chemicals. In addition, EEG studies have observed REM sleep defects and neuro-imaging studies have looked at perfusion defects in relevant brain regions. Rao *et al*.[1] examined liver function tests, serum ascorbic acid and copper levels in 156 schizophrenia patients and compared them with 192 medically ill non psychiatric patients and healthy controls. They did not find any abnormality in liver function test, ascorbic acid and copper levels. Kuruvilla *et al*.[2] assessed serum proteins in 135 patients with chronic schizophrenia patients, 62 with acute schizophrenia and 25 controls. Only chronic patients had trend level increase in alpha 1 and gamma globulin, no difference in albumin, alpha 2 globulin and beta globulin.

Prakash and Sethi[3] assessed the protein fractions of 238 patients with schizophrenia and compared this with 100 controls. They used electrophoretic methods and found that patients had significantly lower albumin levels and higher alpha 2 globulin levels but did not differ in alpha 1 globulin. Narasimha Rao *et al*.[4] studied immunoglobulin profile in 54 patients with schizophrenia. They noticed that there was no significant difference between normal and schizophrenia patients. There was no influence of duration of illness, previous episodes of schizophrenia, family history of schizophrenia, severity of psychosis and duration of hospital stay on immunoglobulin profile.

Kuruvilla *et al*.[5] studied serum prolactin levels in schizophrenics and control subjects to determine the possible correlation of dopaminergic function and schizophrenia. They included 26 patients with schizophrenia and 43 control subjects matched for age and sex. The prolactin values did not significantly differ in these two groups. In addition, the prolactin level showed significant inverse correlation with positive symptoms in female schizophrenic subjects; prolactin level was significantly less in female patients with positive symptoms when compared to female patients with negative symptoms. In male patients also there was a trend in this direction but it did not reach statistical significance. Shrivastava and Tamhane[6] studied serum prolactin level in 20 male and 11 female drug naive schizophrenia patients.

There was a two fold increase in prolactin level but no correlation between baseline serum prolactin level and severity of baseline psychopathology. Authors concluded that baseline prolactin may not be a reliable indicator of psychopathology and prognosis. Singh *et al*.[7] studied the interrelationship of the antioxidant vitamins and antioxidant enzymes, and their overall effect on regulation of oxidative stress induced by haloperidol as compared to olanzapine. They reported that haloperidol caused more oxidative stress along with a significant reduction of important antioxidant parameters. They found Plasma ascorbate to be the chief antioxidant on which the activity of both plasma superoxide dismutase and alpha tocopherol were dependent under oxidative stressful conditions.

Tiwari *et al*.[8] assessed serum acetyl cholinesterase activity in 30 patients with schizophrenia and 30 with depression. A higher level of enzyme activity was noticed in depression than controls. There was a direct relation between duration of illness and increased activity. There was no difference between patients with schizophrenia and controls.

Das *et al*.[9] in their study examined the correlation between REM sleep latency and neuro-cognitive functioning in 15 schizophrenia patients as measured by Wisconsin card sorting test as compared to 15 healthy controls. REM sleep latency period was determined by polysomnography, which included EEG, EOG and EMG. The authors demonstrated a positive correlation between negative symptoms and neuro-cognitive dysfunction and a negative correlation between REM sleep latency and neuro-cognitive dysfunction. The findings suggest that a hyper-cholinergic state exists in schizophrenia as opposed to a loss of cholinergic neurons as seen in dementia and anticholinergic medication might be beneficial in negative symptom schizophrenia.

Gong and colleagues[10] studied the concentration of serum homovanillic acid (HVA), luteinizing hormone (LH), follicle-stimulating hormone (FSH) and testosterone (T) in 20 male schizophrenic patients not taking neuroleptic drugs, 44 treated with neuroleptic drugs and 15 healthy male control subjects. There was no significant difference among the three groups in serum HVA, FSH, LH or testosterone although high concentrations were found in the patients not taking neuroleptic drugs.

Studies examining for brain abnormalities in schizophrenia using neuroimaging techniques have reported Significant hypo-perfusion in frontal as well as temporal areas in childhood onset patients.[11]

FDG-PET study revealed that participants With negative symptoms of schizophrenia had significantly decreased metabolism in all regions of the brain as compared to the positive type. The positive syndrome of schizophrenia was associated with significantly increased glucose metabolism in the medial temporal regions, basal ganglia and left thalamic regions. Hypo metabolism was also noted in the cerebellum.[12]

## BIPOLAR AFFECTIVE DISORDER

Studies in bipolar affective disorder have mainly focused on clinical correlates of serum lithium levels. Srinivasan *et al*.[13] assessed the effect of lithium carbonate on total serum calcium and magnesium in BPAD patients. They included one male and six female patients and noted a significant increase in total serum magnesium levels in manic depressives on recovery from their illness. However, there was no change in serum calcium levels.

Kuruvilla *et al*.[14] reported how the technique of measurement of lithium can be adapted to Indian situation. They reported that it is important to run a quality control sample with each test sample as an independent check on the procedure. They also reported that samples can be stored for five to six days at room temperature without any change in the value.

Prakash and Sethi[15] examined 95 patients who received lithium and investigated the relation between salivary and serum lithium concentrations as the former is convenient to collect. Authors reported highly significant and positive correlations but not of high order. Pandey *et al*.[16] examined serum lithium levels in 18 healthy volunteers after giving 1200 mg of standard and sustained release preparations. There was a significant difference between slow release and conventional lithium after four hours, 12 hours, 20 hours and 24 hours. Authors concluded that high serum lithium level is more likely to occur with slow release preparation.

Khandelwal *et al*.[17] studied the stability in values of serum lithium over a period of eight days. They noticed that values on the first day did not differ from values on other days. They concluded that lithium estimation can be done on a stored sample where facilities do not exist for serum lithium levels.

Shukla *et al*.[18] studied serum potassium changes after direct and modified Electroconvulsive therapy (ECT). They observed that modified ECT can result in increase in serum potassium after three minutes, which is sustained well beyond 10 minutes. Direct ECT caused a quick rise in serum potassium after one minute and then had quick fall.

Geetha *et al*.[19] studied 23 manic depressive psychosis patients and assessed the levels of calcium, magnesium, phosphates and proteins over three months. There was no significant difference in these electrolytes and proteins after lithium treatment. Kuruvilla *et al*.[20] examined a group of 35 patients who had failed to reach therapeutic levels of lithium on treatment with lithium to test the hypothesis whether a dose of 600 mg lithium can predict the daily lithium dose. They reported that it was not useful.

Solanki *et al*.[21] assessed the effect of intravenous sodium valproate in 30 patients with acute manic episodes of bipolar disorder and reported that injectable sodium valproate is a safe and effective mood stabilizer for patients with mania.

## OTHER DISORDERS

Mishra *et al*.[22] studied serum cholesterol, total triglycerides, HDL cholesterol, LDL cholesterol, VLDL cholesterol, free cholesterol and total phospholipids in 36 patients of anxiety and 24 controls. Patients with anxiety disorders had higher Serum triglycerides, VLDL cholesterol and free cholesterol while controls lowered free cholesterol. There was also a significant negative correlation between severity of anxiety and free cholesterol only in females.

Verma and colleagues[23] studied the lipid profile abnormalities as peripheral markers for suicidal risk in 41 patients. Total serum cholesterol, serum triglyceride, LDL levels and HDL levels were lower in suicide attempters but were not significant.

Tharyan and colleagues[24] studied Prolactin levels in 13 patients with epilepsy, 15 patients who underwent bilateral unmodified ECT for the treatment of endogenous depression and 11 patients with hysterical pseudoepileptic seizures. Fifteen age and sex-matched non-epileptic patients with acute medical and surgical illnesses served as controls. They observed significant post-ictal hyperprolactinemia after seizures and after bilateral unmodified ECT but not after hysterical pseudoepileptic seizures or in stressed non-epileptic controls. Thus, a proportionate increase in peak prolactin levels of at least thrice baseline values was found to best differentiate genuine seizures from pseudoepileptic seizures. An MRI study examining acute effects of electroconvulsive therapy concluded absence of detectable brain changes using T_2_-relaxometry technique (Kunigiri *et al*. 2007).[25]

Shah and colleagues[26] compared the serum acetyl cholinesterase level in the patients of opioid dependence and normal volunteers. They included 12 patients with opioid dependence and 12 controls. They noticed a significantly lower level of serum acetylcholinesterase in patients of opioid dependence. Silva *et al*.[27] studied the P 300 component of visual evoked response potential in 24 subjects at high risk for alcohol dependence versus 25 low risk subjects. Subjects were assessed for externalizing symptoms. The P 300 component was measured using target/nontarget visual task. High risk subjects had significantly higher externalizing symptoms scores. P 300 amplitudes at frontal leads were found to be lower in high risk subjects as compared to low risk subjects and this correlated negatively with the ADHD impulsivity sub-score. This study proposes mechanism of central nervous system disinhibition mediating externalizing symptoms and susceptibility to alcohol dependence.

## CONCLUSIONS

In conclusion, review of research studies focusing on biological investigations in psychiatry suggest varied parameters that have been examined with mixed findings. It is possible that uncertainties of these observations are mainly implicated upon the heterogeneity of these disorders. This emphasizes the need for ‘endophenotype-based’ research approaches that might help us to advance our understanding of various psychiatric disorders.
